# Supplementary material for: Exercise and/or Genistein Treatment Impact Gut Microbiota and Inflammation after 12 Weeks on a High-Fat, High-Sugar Diet in C57BL/6 Mice
Source: Nutrients. 2020 Nov 6;12(11):3410. doi: 10.3390/nu12113410 (PMC7694625; doi:10.3390/nu12113410)
Supplement: Supplementary file 1 [file nutrients-12-03410-s001.pdf]

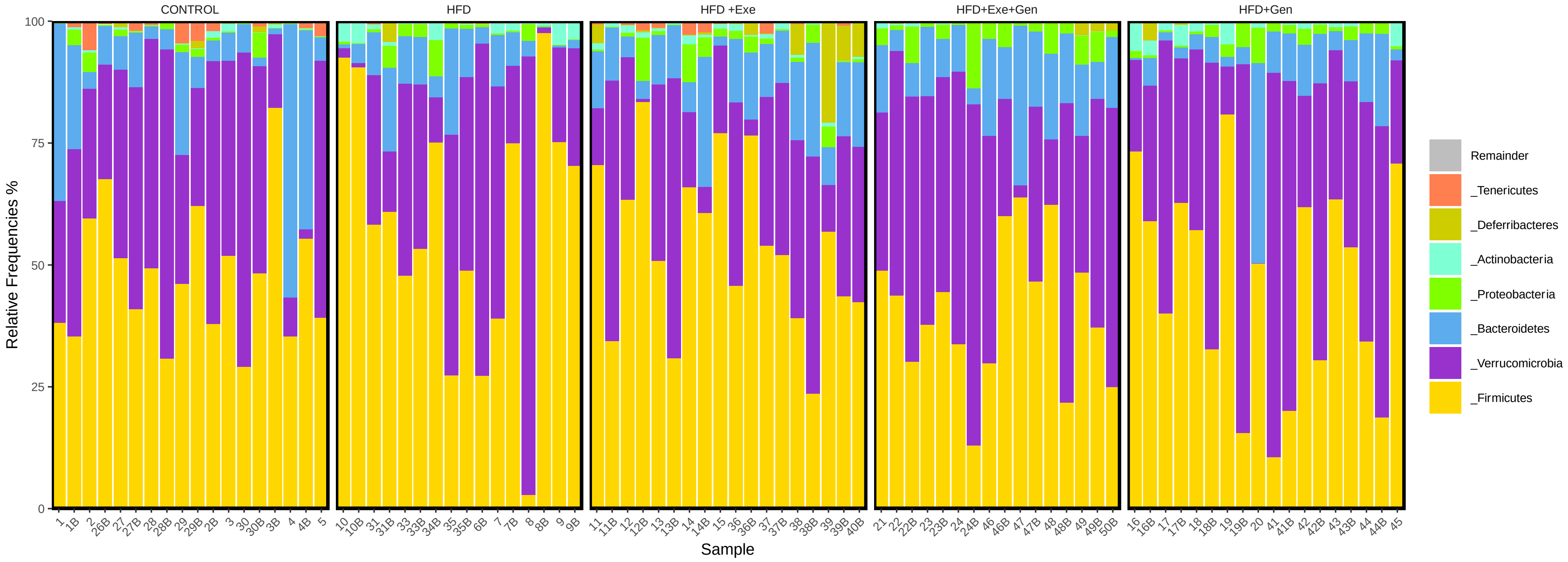

Figure 1. Relative Frequency of Taxa by Treatment Group at the Phylum Level. The top seven phyla, summed across all treatment groups by highest count, are shown in the legend. Taxa present in lower proportions that 10 were summed and placed in the "Remainder" category.
